# Oxidative Stress and Microvascular Alterations in Diabetic Retinopathy: Future Therapies

**DOI:** 10.1155/2019/4940825

**Published:** 2019-11-11

**Authors:** María L. Rodríguez, Salvador Pérez, Salvador Mena-Mollá, M. Carmen Desco, Ángel Luis Ortega

**Affiliations:** ^1^Department of Physiology, Faculty of Pharmacy, University of Valencia, Vicente Andrés Estellés Av. s/n, 46100 Burjassot, Spain; ^2^FISABIO-Oftalmología Médica, Vitreo-retina unit, Bif. Pío Baroja General Avilés s/n, Valencia 46015, Spain

## Abstract

Diabetes is a disease that can be treated with oral antidiabetic agents and/or insulin. However, patients' metabolic control is inadequate in a high percentage of them and a major cause of chronic diseases like diabetic retinopathy. Approximately 15% of patients have some degree of diabetic retinopathy when diabetes is first diagnosed, and most will have developed this microvascular complication after 20 years. Early diagnosis of the disease is the best tool to prevent or delay vision loss and reduce the involved costs. However, diabetic retinopathy is an asymptomatic disease and its development to advanced stages reduces the effectiveness of treatments. Today, the recommended treatment for severe nonproliferative and proliferative diabetic retinopathy is photocoagulation with an argon laser and intravitreal injections of anti-VEGF associated with, or not, focal laser for diabetic macular oedema. The use of these therapeutic approaches is severely limited, such as uncomfortable administration for patients, long-term side effects, the costs they incur, and the therapeutic effectiveness of the employed management protocols. Hence, diabetic retinopathy is the widespread diabetic eye disease and a leading cause of blindness in adults in developed countries. The growing interest in using polyphenols, e.g., resveratrol, in treatments related to oxidative stress diseases has spread to diabetic retinopathy. This review focuses on analysing the sources and effects of oxidative stress and inflammation on vascular alterations and diabetic retinopathy development. Furthermore, current and antioxidant therapies, together with new molecular targets, are postulated for diabetic retinopathy treatment.

## 1. Introduction

Diabetic retinopathy (DR) is a progressive asymptomatic microvascular complication of diabetes that triggers irreversible retinal damage. This common complication is the leading cause of vision loss in working-age adults (20-65 years) and, therefore, economically active people [1–3]. Although DR is not a mortal illness, it leads to emotional distress and reduces daily life functionality, and thus significantly impacts people's quality of life [1]. In addition to the personal and financial implications for patients, DR is associated with significant economic consequences for health public systems because care costs for patients with DR almost double those compared to people without the illness [4, 5].

Approximately one-third of people with diabetes have some degree of DR, and one in 10 will develop a vision-threatening form of the disease [2]. As the worldwide prevalence of diabetes is increasing, the number of people with DR has been estimated to increase from 424.9 million in 2017 to 628 million, respectively, by 2045 [6]. This increase in its prevalence will make DR one of the main public health burdens. This is why early diagnosis of DR and preventing its development are necessary to avoid or delay loss of sight and to reduce the involved costs [1, 3]. Current treatments target late-stage DR when vision has already been significantly affected. Therefore, it is very important to develop novel and more efficient strategies to prevent and treat DR in early stages [1, 4].

## 2. Classification of Diabetic Retinopathy

Progression of DR is related to abnormalities of the retinal microvasculature, including permeability of the blood-retinal barrier (BRB), vascular endothelial cell and pericyte loss, thickening of the vascular basement membrane, subsequent occlusion of capillaries, and retinal neuronal and glial abnormalities [4]. DR is largely asymptomatic, which means that the pathology may be significantly advanced at the time patients become aware of a loss of vision. Therefore, screening to assess the presence and progression of the condition is necessary [7, 8]. The most widely used classification in daily clinical practice to determine the DR development phase is based on the results of the multicentre Early Treatment Diabetic Retinopathy Study (ETDRS) [8, 9]. According to this study, DR may be classified as nonproliferative diabetic retinopathy (NPDR) and proliferative diabetic retinopathy (PDR) based on the presence of visible ophthalmological changes and the manifestation of retinal neovascularisation [10, 11]. When DR affects the macula, it is termed diabetic macular oedema (DME) and is the commonest cause of blindness in diabetic patients [4].

### 2.1. Nonproliferative Diabetic Retinopathy

NPDR is the initial stage of DR, characterized by [12]:

- Microaneurysms are the earliest clinically visible changes in DR, characterised by the dilation of microvessels
- Haemorrhages occur due to the rupture of weakened capillaries. They can be small dots or larger blot haemorrhages in the densely packed deeper layers of the retina
- Hard exudates consist of lipoproteins and other proteins leaking through abnormal retinal vessels. They appear as yellow lipid deposits with a waxy or shiny appearance, and may form a circinate pattern around foci of leaking capillaries and microaneurysms
- Cotton wool spots appear in advanced NPDR stages due to vascular occlusion like white lesions with vague margins [13].

**Figure 1:**
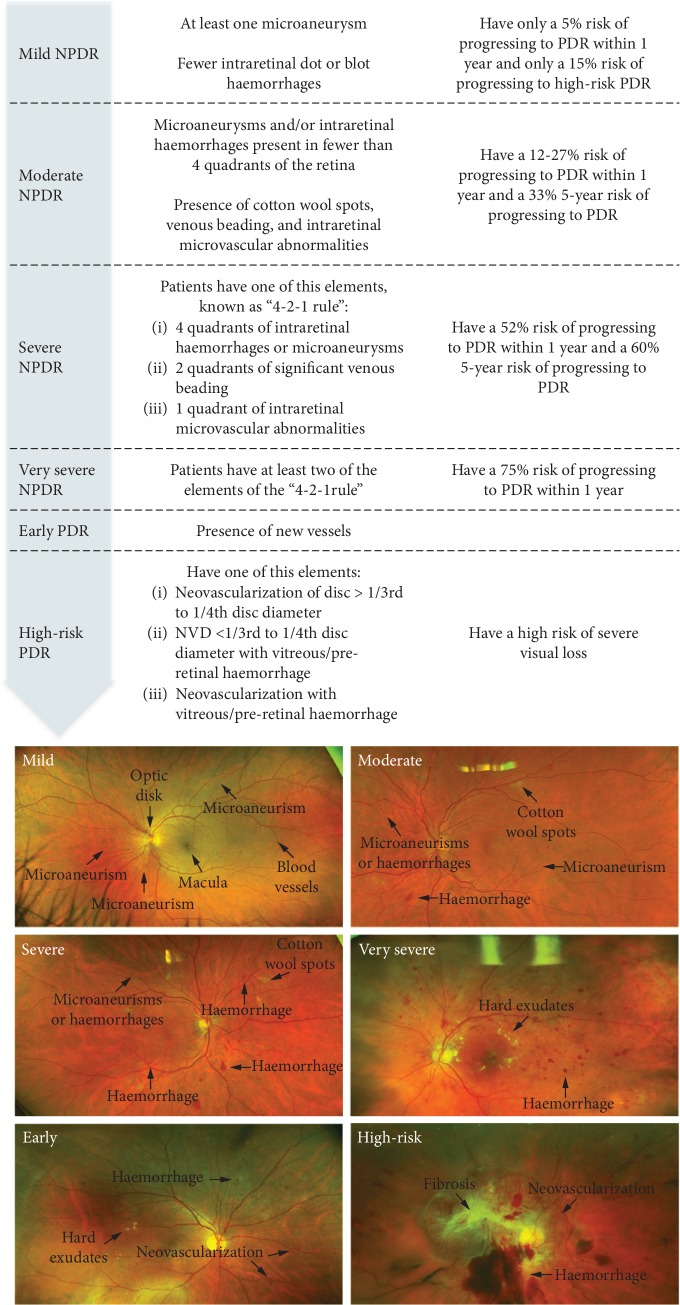
Clinical DR progression and fundus photographs from early background DR to PDR and fibrotic tissue [14–17].

The NPDR can be classified into mild, moderate, severe, and very severe, depending on the number and severity of these changes (Figure 1) [10, 14–17].

### 2.2. Proliferative Diabetic Retinopathy

PDR is the advanced stage of DR and occurs mainly when the occlusion of the blood vessels in the retina is substantial. Ischaemic stimuli induce the neovascularisation process [13]. However, new vessels are leaky, fragile, and misdirected and cause poor retinal blood flow. This might progress to vitreous haemorrhages and, in late stages, may cause tractional retinal detachment and neovascular glaucoma [13]. PDR is classified as low or high risk (Figure 1), but patients with early PDR are at a 75% risk of developing high-risk PDR in 5 years [18].

### 2.3. Diabetic Macular Oedema

DME is a complication of DR caused by the breakdown of outer BRB and the accumulation of intra- and extracellular fluid within the macular retina [12]. DME is the most frequent cause of blindness in diabetes and, although it can occur at any stage, it is commoner in later phases of the disease [8, 19].

## 3. Risk Factors of Diabetic Retinopathy

The risk factors of DR can be divided into modifiable (hyperglycaemia, hypertension, hyperlipidaemia, and obesity) and nonmodifiable (duration of diabetes, puberty, pregnancy, cataract surgery, type of diabetes mellitus, and a family history of DR) [1, 3, 20].

Hyperglycaemia is the major instigator for DR to develop. Two landmark clinical trials have shown the close relation between blood glucose levels and the development and progression of DR: “The Diabetes Control and Complications Trial” (DCCT) and “The UK Prospective Diabetes Study” (UKPDS). The DCCT was published in 1993 using data from 1982 to 1993 in 1,441 subjects with type 1 diabetes mellitus (T1DM). During this clinical trial, intensive hyperglycaemia treatment reduced the incidence of DR by 76% and DR progression by 54% compared to conventional treatment [21]. The UKPDS analysed 5,102 patients with type 2 diabetes mellitus (T2DM) between 1977 and1997 and showed that intensive blood glucose control reduced DR by 25% compared to conventional treatment [22]. Moreover, it has been shown that for every 1% reduction in glycosylated haemoglobin (HbA1c), DR development decreases by 40%, 25% progress to vision-threatening DR, 25% need laser therapy, and blindness in people with diabetes is 15% [3, 23]. It has been recently reported that if the HbA1c level is maintained below 7.6% (60 mmol/mol), it seems to prevent proliferative DR for up to 20 years in T1DM patients [24].

The UKPDS also examined the effect of intensive blood pressure control and showed that after a 9 year follow-up, hypertensive T2DM patients with tight blood pressure control displayed a 34% and a 47% reduction in the DR progression risk and the visual acuity deterioration risk, respectively. It has been also demonstrated that for every 10 mmHg increase in systolic blood pressure, there is a 10% increased risk of early DR and a 15% increased risk of PDR or DME [25, 26]. However, several studies have not found any association between hypertension and incidence or DR progression [3, 27–29].

Regarding hyperlipidaemia and obesity, serum lipids seem to have less influence on the development of proliferative DR or DME [30, 31]. In fact, various studies have reported inconsistent results about the effect of lipids on the development and progression of DR and DME [23, 32–35]. Nevertheless, the DCCT showed that DR severity correlated positively with increasing triglycerides and inversely with high-density lipoprotein (HDL) in T1DM [3, 36].

The UKPDS also reported that DR severity at the time of TDM2 diagnosis was related to impaired *β*-cell function [37]. Indeed the patients with better preserved *β*-cell function were less likely to have DR [37]. Measuring *β*-cell function from fasting glucose and insulin levels showed a relation between retinopathy and the altered function of *β*-cells [38]. Pancreatic *β*-cells use oxidative mitochondrial metabolism as the main source of ATP production, which is required for glucose intake. Interestingly, *β*-cells have a lower antioxidant capacity compared to other metabolic tissues, which places them at risk for oxidative stress induced by mitochondrial oxidative phosphorylation [39]. Indeed oxidative stress has been assumed to be one of the main causes in of *β*-cell failure in many forms of TDM2 [40].

## 4. Diabetic Retinopathy: Aetiology

**Figure 2:**
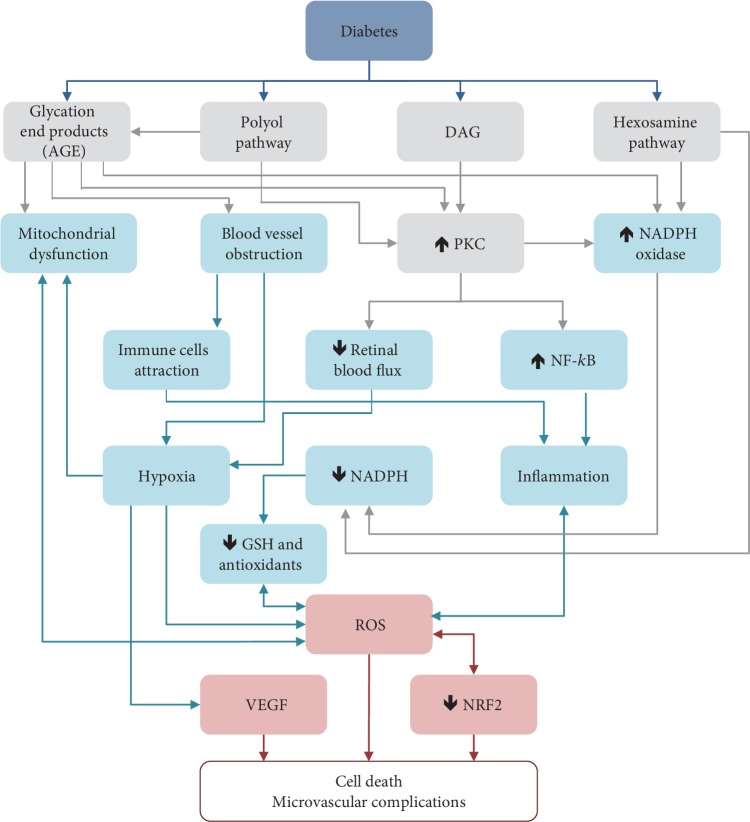
Biochemical pathways involved in ROS and VEGF generation in DR.

DR is a multifactorial disease with a complex aetiology. The exact mechanisms by which high blood glucose levels produce diabetes complications are not altogether clear. Nevertheless, it is known that hyperglycaemia has metabolic effects that induce microvascular damage to the retina [41]. Alterations in biochemical pathways, such as increased flux of advanced glycation end products/receptors (AGE/RAGE), polyol pathway, protein kinase C (PKC) activation, and hexosamine pathway induced by hyperglycaemia, produce oxidative stress (Figure 2) that notably contributes to induced inflammatory intermediate production, causes the rupture of the BRB, pericytes' demise, and increased vascular permeability [42], which lead to progression to advanced DR stages and the development of vascular dysfunctions [41, 43, 44].

### 4.1. Diabetic Retinopathy: Inflammation Relation

Broadly speaking, inflammation is a defensive process mediated by the host immune system in response to injury or stress. Acute inflammation generally results in beneficial outcomes, including tissue defence and repair, while chronic inflammation induces structural and molecular alterations in the retina that often lead to tissue damage and cell death [45]. The inflammatory response of the retinal vasculature can be triggered by different and heterogeneous factors like hyperglycaemia, growth factors, advanced glycation end products (AGEs), high levels of circulating or vitreous cytokines and chemokines, and reactive oxygen species (ROS) [46, 47]. So a large body of evidence confirms chronic inflammation as being critical in DR development, mainly in early stages [41].

The innate immune system recognises two classes of molecules: microbe-specific molecules, termed pathogen-associated molecular patterns (PAMPs), and endogenous stress signals as ROS, known as damage-associated molecular patterns (DAMPs). These molecules are recognised by pattern recognition receptors, such as Toll-like receptors (TLRs) [12, 48]. The interaction of TLRs with PAMPs or DAMPs induces intracellular signalling pathways that subsequently promote the expression of proinflammatory cytokines (i.e., tumour necrosis factor-alpha (TNF-*α*), interleukin-1 beta (IL-1*β*) and IL-6), and monocyte chemoattractant protein-1 (MCP-1) [49]. Adhesion of leukocytes to retinal capillaries (leukostasis), the release of free radicals and proinflammatory cytokines lead to vascular permeability, BRB breakdown, and capillary pericyte loss [42]. In fact, activation of leukocytes and increments in adhesion molecules (ICAM-1 and VCAM-1) have been found to be related to DR progression [50].

Different studies have revealed increased levels of various proinflammatory cytokines, such as vascular endothelial growth factor (VEGF), TNF-*α*, inducible NO synthase (iNOS), cyclooxygenase-2 (COX2), prostacyclin, insulin-like growth factor 1 (IGF-1), nuclear factor kappa B (NF-*κ*B), placental growth factor (PGF), intercellular adhesion molecule-1 (ICAM), IL-1, and IL-6, in the vitreous humours and retinas of diabetic patients and animals [45, 51, 52]. These findings highlight the importance of inflammation in DR. Recent studies have also shown that proinflammatory mediators, such as VEGF, NO, cytokines, chemokines, angiotensin II, the renin-angiotensin system, eicosanoids, and lipids can lead to DME, ischaemia, and neovascularisation [53]. Furthermore, the inhibition or deletion of proinflammatory molecules has been demonstrated to block DR development in animal models [49, 54, 55]. In fact, non-steroidal anti-inflammatory drugs (NSAIDs), anti-VEGF agents, and anti-TNF-*α* agents have been shown to reduce DR progression via their anti-inflammatory properties [18]. Moreover, intravitreal injections of corticosteroids (triamcinolone acetonide, dexamethasone, and fluocinolone) have been shown to reduce vascular permeability, reduce the breakdown of the BRB, inhibit leukostasis, and inhibit VEGF gene transcription and translation in patients with DR [56]. Yet, despite their considerable effectiveness, corticosteroids are associated with a high rate of ocular side effects related to chronic use, including increased intraocular pressure and a higher risk of developing glaucoma and cataract formation, which would accelerate the need for cataract surgery in up to one third of patients with DR and DME [56, 57]. For this reason, the use of intravitreal corticosteroids in clinical practice is currently reserved as a second-line treatment in patients with previous cataract surgery [49].

### 4.2. Diabetic Retinopathy: Oxidative Stress Relation

Under physiological conditions, the production of free radicals, based mainly on reactive nitrogen species (RNS) and oxygen ROS (e.g., superoxide anion (O_2_^·−^), hydroxyl radical (HO^·^) and hydrogen peroxide (H_2_O_2_)), is normal and inevitable. In fact, low/moderate levels of free radicals are needed for physiological activities because they act as messengers in redox signalling to promote cell metabolism, proliferation, differentiation, immune system regulation, and vascular remodelling. To control their levels, cells use enzymatic and nonenzymatic antioxidant defence systems. However, a disruption in redox homeostasis towards excess ROS induces oxidative stress, which can damage biological macromolecules like proteins, lipids, carbohydrates, and nucleic acids [58–60].

In recent decades, numerous studies have highlighted the high level of ROS in diabetic retina and its role in cellular signalling alteration, which contribute to retinal cell damage and finally to DR pathogenesis development [61].

#### 4.2.1. ROS Production through Advanced Glycation End Products

Due to hyperglycaemia, nonenzymatic binding of glucose to macromolecules occurs (amino acids in proteins, lipids, and nucleic acids), which leads to the formation of AGEs [62]. AGEs bind to their receptors, known as RAGE (receptor for AGE), in different cell types, such as macrophages and vascular endothelial and vascular smooth cells, which affects retinal functionality.

Diabetic patients accumulate AGEs in circulation due not only to their inefficient renal clearance, but also to their insufficient degradation by ubiquitination or autophagy. Kadarakis et al. identified three harmful mechanisms of action induced by prolonged AGEs exposure, which causes irreversible tissue damage [63], including diminished vessels elasticity owing to AGEs modification on extracellular matrix structural proteins, activation of different AGE/RAGE cellular signalling pathways, such as mitogen-activated protein kinases (MAPKs) cascades that induce the translocation of NF-*κ*B with disorders in cell function [64], and glycation of intracellular proteins and lipids that is capable of changing their functionality and ROS production [63]. AGE accumulation may induce retinal vessel obstruction and lead to ischaemia and activate intracellular signalling, such as MAPK/NF-*κ*B. These processes provoke hypoxia, the production of cytosolic ROS, and the reduction of the antioxidant defence system and glycation of protein and nucleic acids of the mitochondria, as well as defects in the electron transport chain that trigger the cascade of inflammatory signals by inducing a “self-propagating” vicious cycle of free radicals [62, 65, 66].

Hyperglycaemia increases triose phosphates, dihydroxyacetone phosphate, and glyceraldehyde-3-phosphate, which lead *α*-ketoaldehyde methylglyoxal (MGO), a low-molecular-weight compound precursor of AGEs, which is cytotoxic and highly reactive with DNA, RNA, and proteins. Under physiological conditions, glyoxalase I and II detoxify MGO. However, in diabetic patients, AGE accumulation can induce apoptosis of pericytes due to glyoxalase I inactivation in a peroxynitrite-dependent mechanism [67]. The inactivation of glyoxalase I and MGO accumulation reduce retinal pigment epithelial cell viability via endoplasmic reticulum stress-dependent ROS production and mitochondrial dysfunction [68]. MGO administration mimics some characteristic aspects of the phenotype of DR development, such as microglial activation, vascular damage, and neuroretinal dysfunction, particularly after its administration in the absence of hyperglycaemia [69].

The increasing production of O_2_^·−^ mediated by AGEs in mitochondria under hyperglycaemic conditions can produce an early imprint in the cells of the vasculature, which may permit the development of microvascular abnormalities, even with good glycaemic control. This permanent dysfunction, known as “metabolic memory,” is a severe condition observed in DR and is responsible for the endothelium's failure to preserve vascular homeostasis [70].

The metabolic memory phenomenon could explain DR development despite patients undergoing intensive glycaemic control as there would be a reprogramming in initial disease stages when hyperglycaemia would be linked to self-supported oxidative stress, nonenzymatic glycation of proteins, epigenetic changes by DNA methylation, posttranslational histone modifications or microRNA alteration, and chronic inflammation in early phases that would produce irreversible changes in gene expression and mitochondrial function [71].

#### 4.2.2. ROS Production through the Polyol Pathway

Under normal conditions, glucose is metabolised through the glycolytic pathway. However, on the hyperglycaemia polyol pathway, it is activated and glucose is reduced to sorbitol by aldose reductase, which is converted into fructose. Polyol pathway activation in diabetes can produce oxidative stress via two mechanisms. On the one hand, sorbitol production by aldose reductase increases NADPH consumption to compromise the availability of reduced glutathione (L-*γ*-glutamyl-L-cysteinyl-glycine (GSH)) [72]. GSH is the most abundant nonprotein thiol and natural antioxidant in eukaryotic cells. Besides protection against free radicals, GSH performs other important cellular functions, such as DNA synthesis and cell proliferation [73]. In the retina, GSH plays a critical protector role in reducing the oxidative stress which the retina is subjected to on a daily basis [74]. On the other hand, fructose synthesised from sorbitol by the polyol pathway is phosphorylated to fructose-3-phosphate which, in turn, is broken down to 3-deoxyglucosone, a glycosylating agent that can contribute to the formation of AGEs [75].

Furthermore, sorbitol production and accumulation under hyperglycaemia may have another adverse effect. Sorbitol is a strong hydrophilic alcohol with little ability to diffuse through cell membranes, which produces cellular hypertonicity that causes osmotic damage and cell demise in retinal capillaries [76, 77].

Despite the polyol pathway having been related to DR, clinical trials using aldose reductase inhibitors have been inconclusive [78, 79] and show that further work is needed to understand the importance of the polyol pathway in DR development.

#### 4.2.3. ROS Production through PKC Activation

PKC is a family of cAMP-dependent protein kinases with multiple isoforms. PKC-*α*, PKC-*β*1, PKC-*β*2, and PKC-*γ* are activated by phosphatidylserine, calcium, and diacylglycerol (DAG) or by phorbol esters. PKC-*δ*, PKC-*ε*, PKC-*θ*, and PKC-*η* are activated by phosphatidylserine, DAG or phorbol 12-myristate 13-acetate (PMA), but not by calcium, while PKC-*ζ* and PKC-*ι*/*λ* are not activated by calcium, DAG, or PMA [80].

Different PKC isoforms are activated by hyperglycaemia through DAG induction, or indirectly through AGE/RAGE and polyol pathways by oxidative stress [81, 82]. Oxidative activation is possible due to residues that make the regulatory domain susceptible to redox modulation in the PKC structure [83].

PKC contributes to the damage of retinal capillaries by activating NAD(P)H-oxidase (NOX). The produced O_2_^·−^ [84] interact with NO and form peroxynitrite which can, in turn, oxidise histone 4B (H4B) to result in endothelial NOS uncoupling and perxynitrite production with subsequent endothelial dysfunction [85, 86].

PKC regulation by an increase in oxidative and nitrosative stress can contribute to the redox mechanism-mediated signalling events of the DR pathogenesis in many cellular functions, such as survival, growth and proliferation, migration, and apoptosis. In fact PKC-*δ* is implicated in the acceleration of the apoptosis of capillary cells by increasing the transcriptional expression of the Scr homology-2 domain that contains phosphatase-1 (SHP-1) which, in turn, leads to the dephosphorylation of platelet-derived growth factor receptor-*β* (PDGFR-*β*) [87], critical for retinal pericyte survival. This inactivation of PDGFR-*β* results in pericyte loss, which leads to the formation of microaneurysms and the attraction of leukocytes, which are early histopathological signs of DR [88].

Although initial clinical trials have revealed the PCK*β* inhibitor, ruboxistaurin, as an interesting therapy to treat DR, the global benefit seems to be minimum, as indicated by the European Medicines Agency [89]. However, the role of the different PKC isoforms in DR is not completely understood and offers new therapeutic targets to treat this irreversible pathology.

#### 4.2.4. ROS Production through the Hexosamine Pathway

Another biochemical pathway is induced in response to chronic high glucose levels called the hexosamine pathway. Chronic hyperglycaemia leads to enhanced influx through fructose-6-phosphate conversion into glucosamine-6-phosphate (GlucN-6-P), catalysed by glucosamine:fructose-6-phosphate-aminotransferase (GFAT). GlucN-6-P is rapidly converted into uridine-5-diphosphate-N-acetylglucosamine (UDP-GlucNAc), a precursor of glycoproteins, glycolipids, proteoglycans, and glycosaminoglycans with the enzymatic action of O-N-Acetyl-GluN transferase (OGT) [90].

Similarly to the polyol pathway, Horal et al. showed that glucose and GlucN-6-P inhibit glucose-6-phosphate dehydrogenase (G6PD) and decrease NADPH-dependent GSH production to lead to H_2_O_2_ accumulation [91]. Furthermore, overexpression of GFAT or administration of glucosamine can increase H_2_O_2_ levels in a dose-dependent manner, which supports the important role that oxidative stress plays in the hexosamine pathway [92]. Indeed overactivity of OGT has been associated with alterations in the gene expression of TGF-*β* through a mechanism dependent on p38 [93]. In turn, TGF-*β* may induce mitochondrial ROS production, NOX activation, and suppression of the antioxidant system [94, 95]. Antioxidant treatment with N-acetyl cysteine (NAC) prevents some adverse effects caused by the activation of the hexosamine pathway [92].

Activation of the hexosamine pathway is implicated in the apoptosis of retinal capillary cells and limits pericyte proliferation in diabetes [61]. Hence, the importance of understanding how hyperglycaemia alters different pathways through changes to only a few essential cellular elements is critical for us to understand the main role of oxidative stress in DR development.

## 5. NRF2 in Diabetic Retinopathy

### 5.1. Overview of NRF2

Nuclear factor erythroid 2-related factor 2 (NRF2) is a redox-sensitive basic leucine zipper region transcription factor and a member of the Cap ‘n' Collar family of regulatory proteins, with approximately 605 amino acid residues and seven functional domains, Neh1–Neh7, crucial for their self-regulation and activity [96]. NRF2 functional activity depends on the cellular distribution between the nucleus and the cytoplasm. Under physiological conditions and with a correct balance between antioxidant and prooxidant species, NRF2 is located in the cytoplasm attached to Keap1 and Cullin3 and establishes an inhibitory complex for the ubiquitination and proteasome degradation of NRF2 [97]. Oxidative stress induces conformational modifications in Keap1 cysteine residues, which promote the breakdown of the NRF2-Keap1-Cullin3 complex. Free cytosolic NRF2 is translocated to the nucleus, forms a heterodimer with transcription factor Maf, and binds to antioxidant response element (ARE) sequences to result in the expression of phase II detoxifying and antioxidant genes [98]. These genes include heme oxygenase 1 (HO-1), NAD(P)H dehydrogenase (quinone) 1 (NQO1), thioredoxin reductase (TrxR), peroxiredoxins (Prxs), superoxide dismutase (SOD), catalase (CAT), GSH peroxidase (GPx), GSH reductase (GR), and GSH S-transferase (GST) and glutamate-cysteine ligase (GCL), which rapidly clear free radicals and play a critical defensive role in cell homeostasis [99].

In the presence of NADPH, HO-1 catalyses the oxidative cleavage of the heme group from haemoglobin to biliverdin, ferrous iron (Fe^2+^), and carbon monoxide (CO). NQO1 catalyses the two-electron reduction of quinone to the redox-stable hydroquinone by preventing free radical formation from quinone derivatives. It can reduce O_2_^·−^, but less efficiently than SOD. The Trx system, which includes Trx, TrxR, and Prx, reduces disulphide bridges of various proteins and removes O_2_^·−^ or peroxides.

O_2_^·−^ is neutralised by SOD into H_2_O_2_, which is degraded by CAT in peroxisomes or by GPx in cytosol or mitochondria to O_2_ and H_2_O. For these functions, GPx needs to oxide GSH to GSSG (glutathione disulphide, the oxidised form), which is renewal by GR to GSH using NADPH as a coenzyme. GST detoxificates reactive electrophillic compounds through a conjugation with GSH. Finally, GCL is the limiting enzyme of GSH synthesis and catalyses glutamate-cysteine binding [100, 101].

### 5.2. Role of NRF2 in Endothelial Dysfunction of Diabetic Retinopathy

NRF2 plays a key role in retinal vasculature protection from ROS-associated injury. In diabetes, NRF2 increases in the retina. However, its nuclear levels lower and compromise the antioxidant defence system [102]. Using an animal diabetic model based on streptozotocin administration, a deficient transport of NRF2 to the nucleus has been reported due to increased Keap1 expression [103]. Activity of NRF2-associated antioxidant enzymes is reduced in *in vitro* models [104]: HO-1 in animal diabetic models [103] and SOD, GR, GPx, and CAT in patients with diabetes [105, 106]. Reduced antioxidant capability in diabetic patients, together with the oxidative environment generated by hyperglycaemia, notably increases the risk of developing DR.

Several animal models of diabetes report alteration to the NRF2-Keap1 complex in endothelial cells exposed to high glucose levels [107]. Indeed NRF2 deficiency leads to unchecked NOX2 activation and triggers ROS accumulation [108]. Interestingly in initial diabetes phases, NOX may provide a source to activate NRF2-mediated antioxidant gene expression to modulate redox homeostasis. NRF2 also promotes reparative angiogenesis in later diabetes phases through NOX2 regulation in an oxygen-induced retinopathy model [109]. However, chronic hyperglycaemia increases ROS and saturates the NRF2-ARE defence pathway, leading to more ROS production [110].

NRF2-/- mutant mice have been used in diabetes studies to clarify the role of the antioxidant target genes of NRF2 in the retina. *In vivo* models of high-fat diet reveal endothelial dysfunction to be more severe in NRF2-/- mice than in wild-type mice [111]. These results suggest that endothelial protection in response to hyperglycaemia is caused by the upregulation of GCLc, HO-1, and NQO1 in other diabetic complications models [111]. Moreover, lack of NRF2 under hyperglycaemic conditions brings about a decrease in retinal ganglion cells due to the reduced capacity to respond to oxidative stress [112].

Moreover, NRF2 plays a pivotal role in mitochondrial homeostasis [113, 114]. Diabetes alters the membrane potential of retinal mitochondria and NRF2 prevents this mitochondrial injury by increasing manganese SOD (MnSOD) expression [115]. In addition to mitochondrial integrity, NRF2 controls mitochondrial biogenesis, which is also impaired in diabetes [116] by modulating one of its downstream targets, NRF1, a major transcriptional regulator of mitochondrial biogenesis retinal cells [117, 118]. The NRF2-Keap1 signalling pathway shows a direct crosstalk with mitochondrial ROS. NRF2 activation decreases ROS production in cytosolic and mitochondrial NOX by inducing HO-1 and NQO1 [119].

There is plenty of scientific evidence to indicate that NRF2 regulates the inflammatory response by modulating the expression of both NF-*κ*B and COX2. Lack of NRF2 activity triggers an increase in proinflammatory cytokines due to the induction of NF-*κ*B, which is associated with capillary cell apoptosis in diabetes via the overexpression of proapoptototic Bax or TNF-*α* [120–122]. NF-*κ*B could interact with Keap1 by promoting the degradation of Keap1 and the nuclear translocation of NRF2, a mechanism observed in DR [123]. In an experimental model of streptozotocin-induced diabetes, an anti-inflammatory action on COX2 has been observed via the activation of the NRF2/HO-1 signalling pathway, which protects against neuron damage in diabetes [124].

## 6. VEGF and Neovascularisation in Diabetic Retinopathy

The retina is one of the most metabolically active tissues of the organism, which makes it extremely sensitive to alterations in oxygen levels [125]. Notwithstanding, retina cells are constantly exposed to the effects of ROS from both the external environment (ultraviolet radiation, metals, or cigarette smoke) and endogenous metabolism (alterations in respiration, mitochondrial, or viral infections) [126].

Under physiological conditions, oxygen and nutrients are supplied to the optic nerve from the choroidal blood flow [127], and epithelia and retinal vessels regulate a stable environment needed for appropriate retinal function. Endothelial cells have tight junctions which, together with pericytes and neuronal and glial cells, transform the retina into a privileged place similarly to the central nervous system or testis as this BRB protects the neural retina from the leakage of circulating blood toxins. However, nonhomeostatic increases in glycaemia and ROS overproduction trigger phenotypical changes in vascular terms, including retinal ischaemia, high permeability, and retinal neovascularisation induced by the overproduction of VEGF and DME [128–130]. The formation of new immature fragile vessels on the surface of the retina in an advanced stage generates vitreous haemorrhages and even tractional retinal detachments, which lead to severe and irreversible loss of vision [131, 132].

The VEGF family consists in a large family of members responsible for the maintenance and growth of new blood vessels by playing a critical role in retinal homeostasis but are also involved in the pathological angiogenesis observed in diseases like DR. VEGFA, usually referred to as VEGF, is the most important proangiogenic member of this family. In fact, it has been shown that the release of VEGF by the retinal pigment epithelium plays a critical role for correct choriocapilaris development [133, 134], and also in the maintenance of correct retina functionality in adult individuals [135]. During DR development, retinal hypoxia is a common factor in the development of pathological angiogenesis [135], which is associated with high levels of VEGF, among other causes [136].

Although different stimuli can increase VEGF production, oxidative stress plays a key role by increasing growth factor levels and inducing DR evolution to pathological advanced stages. Using retinal pigment epithelial cells and the Ins2Akita mouse model, Simão et al. showed that high-glucose concentrations modulate the expression of different isoforms of VEGF by ROS production [137]. Meanwhile, VEGF165b levels, an endogenous splice variant of VEGF with antiangiogenic properties [138], have been reduced and VEGF production has been increased [137]. As the balance of isoforms is lost under the pathological condition, VEGF splicing can be a good target to avoid neovascularisation during DR progression [139].

Another family member, VEGF-B, impairs insulin sensitivity in rodent models of T2DM and its signalling depletion improves glucose tolerance, preserves the pancreatic islet architecture, improves *β*-cell function, and ameliorates dyslipidaemia [140]. For all these reasons, VEGF-B signalling inhibition has been suggested for T2DM treatment [140]. However, *in vitro* studies done with human retinal endothelial cells have also shown that high glucose levels lower VEGF-B expression [141] and it was not possible to validate this effect using vitreous fluid samples from patients with PDR [142]. In the present day, the main role of VEGF-B in DR evolution remains controversial and further research is needed.

High glucose levels, AGEs, and VEGF production have been directly related both *in vitro* and *in vivo* [63, 143] via extracellular signal-regulated kinases (ERK) activation pathway [63]. Furthermore, AGEs reduce pigment epithelium-derived factor (PEDF) expression, a known anti-inflammatory and antiangiogenic actor involved in DR development via ROS generation [143], NF-*κ*B activation, the proinflammatory environment with cytokines release, such as IL-6 and TNF-*α*, adhesion molecules, and growth factors, i.e., insulin-like growth factor 1 and TGF-*β* [63].

Wautier et al. demonstrated that gp91phox, the central subunit of NOX2, is activated by AGEs by increasing O_2_^·−^ production [144]. Moreover, ROS derived from gp91phox are associated with VEGF signalling activation and lead to neovascularisation under ischaemic conditions [145].

Alterations to the retinal blood flow of diabetic patients were suggested in the 1930s [146]. Nowadays, retinal hypoperfusion has been demonstrated in animal models and patients as one of the first alterations observed in early DR [147]. This effect is attributed not only to the acceleration of apoptosis of capillary cells and microaneurysms formation and fragile neovascularisation by VEGF production but also by vascular electrotonic control loss. The retina lacks autonomic innervation, so effective blood flow autoregulation is essential to maintain tissue functionality [148, 149]. In this way, the electrotonic architecture of the retinovasculature plays a main role as it forms the infrastructure needed to spatially integrate voltage-changing vasomotor signals [150–152]. Moreover, loss in blood flow autoregulation in early DR is attributed to alterations in the vascular electrotonic transmission signals generated along the vascular network from early disease phases [150–152]. Electrotonic transmission within retinal microvessels becomes markedly impaired [153], and the ability of the retina to autoregulate blood flow is lost in early DR [154–156]. The large number of abluminal pericytes with similar contractile characteristics to myocytes and their high interconnectivity by gap junctions indicate how the contraction control modulates tissue perfusion [151, 157, 158]. Recently, Shibata et al. have correlated this effect to the activation of both PKC and VEGF. Moreover, the use of nondiabetic microvessels under normoglucaemic condition has demonstrated that transmission inhibition induced by VEGF is independent of glucose concentrations, which is interesting to understand damage to electrotonic transmission in nondiabetic pathologies in which VEGF production is induced, i.e., by hypoxia [159].

The retina is the tissue with the highest concentration of polyunsaturated long-chain fatty acids (PUFAs) (20%). These molecules play essential roles in the architecture and function of the retina [160]; for example, lipid peroxides, such as 4-hydroxinonenal, alter lysosomal functionality by generating lipofuscin accumulation [161]. In retinas with an intact BRB, plasma lipoproteins may be largely irrelevant. However, major effects become operative after this barrier is impaired in diabetes, which leads to lipoprotein extravasation, which is well correlated with DR severity. The finding of plasma lipoprotein oxidation in diabetic retinal tissue prior to the onset of clinical evidence is consistent with its role in promoting early DR [162]. Moreover, the plasma lipoproteins altered by oxidation and glycation after leakage into the retina contribute to spread DR [163], and the alteration of glycaemic and lipid metabolism lowers the *α*-ketoglutarate levels in the cells of the retina, stabilises hypoxia inducible factor 1*α* (HIF-1*α*) levels, stimulates VEGF secretion, and induces angiogenesis [164].

## 7. Present and Future Therapeutic Targets

DR is the most important cause of sight loss at the working age [165, 166]. By 2015, DR was the fifth leading cause of such damage with 2.6 million patients, and the Vision Loss Expert Group estimated this number will increase to 3.2 million in 2020 [166].

DR represents a high social and health cost. Despite the increasing prevalence of the disease, it is difficult to estimate its real economic burden. Recently, it has been estimated that the annual medical cost for the Spanish National Health System to treat diabetes is €5.1 billion, of which €1.5 billion are due to diabetes-associated complications. To these expenses, we must add work productivity loss, which comes to €2.8 billion [167], and that the affected people work, which is a high productive period of their lives. Although DR is a chronic disease with no impact on the mortality of diabetic patients, it affects their quality of life in both psychological and functional terms.

The current treatment of early DR includes tight glycaemic control. However, as indicated above, despite strictly controlling glycaemia, irreversible changes may have occurred before diagnosing diabetes due to “metabolic memory.” This may be why treatment begins in most cases when patients already notice obvious symptoms of damage to the retina.

The treatment indicated for severe or preproliferative/proliferative DR stages is photocoagulation with argon laser and is immunotherapy through the intravitreal injection of anti-VEGF associated, or not, with focal laser for diabetic DME. Although newer anti-VEGF drugs have been shown to be cost-effective when used either as a monotherapy or combined with focal laser [2], these therapeutic approaches have serious limitations in terms of their use: uncomfortable administration to patients, long-term side effects, the economic cost, and poor therapeutic effectiveness of some administration protocols. Although these therapies delay DR progression and loss of sight, damage to retinal blood vessels and neuronal cell functions is irreversible. Therefore, new targets and therapies are needed to prevent or delay DR development.

NOX are a main enzymatic source of ROS and are directly related with promoting pathological neovascularisation in the retina by hyperglycaemia [168]. To date, the isoforms of the NOX family which have been best studied in terms of their relation with DR are NOX1, NOX2, and NOX4, while different inhibitors have been shown to prevent DR development [169].

Diphenyleneiodonium and apocynin are inhibitors of NOX. Although their therapeutic effects include lowering ROS and VEGF levels, both show NOX-independent effects that lower ROS levels. Thus, it is difficult to establish their therapeutic effect on DR based exclusively on NOX inhibition [169]. The development of specific NOX inhibitors may improve DR treatment [169].

Triamcinolone acetonide, dexamethasone sodium phosphate, and fluocinolone acetonide are corticosteroids used to treat DR, either alone or combined with laser therapy or anti-VEGF injections. The main effect of the intravitreal administration of these compounds is to reduce inflammation and reestablish the disrupted BRB [42]. In addition, the anti-inflammatory effect of corticosteroids is able to reduce the expression of VEGF induced by hypoxia [170]. For this reason, their administration is a good alternative to anti-VEGF drugs to treat diabetic DME for whom anti-VEGF therapies are contraindicated, e.g., patients with coronary diseases [42].

To date, the role of dyslipidaemia in DR development remains relatively unexplored and is controversial. A recent systematic review and meta-analysis found no differences in triglycerides, serum total cholesterol, and high-density lipoprotein cholesterol among patients with or without DR [35]. However, many studies have shown how using lipid-lowering medication, such as statins [171–173] or fenofibrate [35, 173], reduce DR progression. Recently, a study used an animal model and described how fenofibrate inhibited the expression of NF-*κ*B and inflammatory chemokines, and also reduced oxidative damage in lipids, DNA, and proteins in diabetic retinas [174]. So, it would seem obvious that the action of lipid-lowering drugs involved in controlling diabetes progression deserves further research.

Since the US Food and Drug Administration published recommendations about cardiovascular safety for new diabetes therapies in 2008 [175], different molecules have emerged as effective treatments for T2DM. Glucagon-like peptide 1 receptor agonists (GLP1RAs) have been incorporated by the European Association for the Study of Diabetes as a safe treatment. Furthermore, preclinical studies show the benefits of GLP1RAs on diabetic vascular complications such as DR [176]. Actually, GLP1R activation reveals a plethora of effects independently of glycaemia homeostasis by diminishing the pernicious consequences of high glucose exposure on the retina, such as neurodegeneration [177], inflammation [178], oxidative stress [179], BRB breakdown [180], or angiogenesis [178]. An important target of GLP1R activation is the AKT pathway, which is essential for retinal neuroprotection in early DR development [178]. AKT phosphorylates a myriad of proteins (E2 ubiquitin ligases, transcription factors, protein and lipid kinases, metabolic enzymes, etc.) and the main role of AKT in DR has been revealed. For example, the first AKT substrate to be reported was GSK3 [181]. GSK3 inactivation by AKT-phosphorylation has been shown to be a clue in the regulation of transcription factors like HIF1*α* or NRF2 [182], two essential actors for DR development.

Despite the advances made in DR therapy, many patients still reach advanced stages of the disease. Therefore, it is necessary to research in-depth to identify new therapeutic approaches.

### 7.1. Polyphenols and Diabetic Retinopathy

Antioxidants are defined as compounds that can delay, inhibit, or prevent oxidative damage. Polyphenols are the most abundant antioxidants in our diet [183]. Natural polyphenols are plant secondary metabolites abundant in fruits, vegetables, and whole grains and also in the foods and beverages derived from them, such as chocolate, wine, olive oil, or tea. They are derived from the shikimate-derived phenylpropanoid and/or the polyketide pathway(s), and their basic structure contains two phenolic rings or more [184].

A review about polyphenols against diabetes complications with more than 400 natural products was published in 2019 [185], and an excellent work that focused on the complications of age-related eye diseases can be found in Bungau et al. [186]. Indeed, several publications have suggested diabetes mellitus-induced retinopathy protection for various polyphenols, including green tea polyphenols, resveratrol, curcumin, quercetin, and cocoa polyphenols.

Resveratrol (3,4′,5-trihydroxy stilbene; RESV) is the most popular and studied polyphenol. Oral administration of RESV protected streptozotocin-nicotinamide-induced diabetic rats by attenuating hyperglycaemia-mediated oxidative stress and inflammatory cytokines via NRF2-Keap1 signalling [187]. In different experimental models, RESV is able to stimulate the activity of SOD, CAT, and GPx, increases GSH levels [184, 188, 189], and suppresses endothelial NOS activity in blood and the retina [190] with a reduction in ROS and nitrosative stress.

This strong RESV antioxidant capacity can be due to the inhibition of the NF − *κ*B pathway [191]. In a high-glucose environment, ROS decrease the phosphorylation of the 5′-adenosine monophosphate-activated protein kinase (AMPK), a regulator of histone deacetylase Sirtuin 1 (Sirt1). NF-*κ*B inhibition may be induced by different processes: (a) deacetylation of RelA/p65, which allows I*κ*B to retain NF-*κ*B in the cytosol; (b) epigenetic modifications to chromatin that block NF-*κ*B binding to DNA, (c) MAPK-p38 regulation. However, SIRT1 activation by RESV promotes RelA/p65 deacetylation and suppresses NF-*κ*B activation [189, 192, 193].

David Sinclair, famous for discovering the antiageing effect of RESV and sirtuins (deacetylases), showed that low to moderate doses of RESV (25–30 mg/kg/day) activate SIRT1 after increasing the cellular levels of NAD^+^ and deacetylate LKB1 and lead to the phosphorylation and activation of the AMPK pathway. However, at high doses of RESV (approx. 10-fold), AMPK activation is SIRT1-independent and inhibits cAMP-degrading phosphodiesterases [194]. These results are an example of the importance of evaluating the experimental doses used.

The natural polyphenol pterostilbene is a structural analogue of resveratrol with beneficial biological effects that outperform resveratrol [184]. This polyphenol has shown great effectiveness against different pathological actions of diabetes. Pterostilbene lowers the blood glucose level in hyperglycaemic rats [195] and improves carbohydrate metabolism [196]. In streptozotocin-treated INS-1E rat pancreatic *β*-cells, pterostilbene administration induces significant NRF2 activation and increases the expressions of HO-1, SOD, CAT, and GPx [197]. Furthermore, Sireesh et al. showed that in diabetic mice treated with pterostilbene, the expression of NRF2 mRNA and its subsequent targets increase [198]. Moreover, stilbene triggers a high phospho-NRF2 level in nuclear fractions, as well as lower levels of inducible NOS [198]. *In vitro* studies in MIN6 cells have reported antiapoptotic properties by lowering the BAX/Bcl-2 ratio, annexin-V positive cells, and caspase-3 activity associated with NRF2 activation [198].

Other polyphenols from cocoa, mainly epicatechin, prevent oxidative stress in ARPE-19 exposed to glucose [199]. In this work, the authors postulate that treatment with polyphenol-enriched cocoa reduces ROS, and consequently, improves the NAD^+^/NADH ratio by increasing SIRT1 activity and abolishing NF-*κ*B binds to the proinflammatory gene promoter. This hypothesis was validated by siRNA and inhibitors to SIRT1 [200]. As a result of preventing NF-*κ*B activity, the expression of TNF-*α*, IL-6, and COX2 is diminished and the apoptosis rate of retinal cells significantly lowers [191, 201, 202].

Besides antiapoptotic, anti-inflammatory, and antioxidant effects, polyphenols, such as quercetin, chlorogenic acid, caffeic acid, kaempferol, luteolin, (+)-catechin/(-)-epicatechin, betaglucogallin, gallic acid, p-coumaric acid, ferulic acid, quinic acid, cyandin -3-glucoside, peonidin-3-glucoside, ferulic acid, and tocopherol, inhibit aldose reductase activity and the polyol pathway [203, 204].

Hyperglycaemia downregulates autophagy and accumulates P62/SQSTM1 due to lysosomal dysfunction, which induces apoptosis. Treatment with EGCG [205], RESV [206], and a large number of polyphenols including quercetin, silibinin, catechin, and curcumin can reestablish autophagy to remove the aberrant protein aggregates that protect against cell death [207].

Diabetes and ROS induce local hypoxia with upregulated mRNA expression levels of proapoptotic proteins, HIF-1*α*, and AMPK phosporylation, which activate cell death VEGF and production [203, 204]. A protective effect for diabetic retinopathy has been observed with resveratrol [190], epigallocatechin gallate [208, 209], curcumin [210], and quercetin [211, 212], among others. The common factors of them all are antioxidant capacity against oxidative stress after hypoxia [213, 214] and their good capacity to stop the pathological angiogenesis [135] by lowering VEGF levels [136]. So, it has been suggested that polyphenolic administration can protect against neoangiogenesis developing in DR.

The study of new therapeutic developments is important as the clinical trials which have used anti-VEGF therapies indicate that a high percentage of patients develop resistance to anti-VEGF treatment and adverse effects [206].

## 8. Conclusions

Avoiding natural progression from early stages should be the main objective to fight against DR. Modern therapies focus on late DR stages and are able to delay loss of sight by temporarily preventing the formation of abnormal retinal vessels. However, they are unable to recover damaged neural tissue or reestablish visual acuity at 100%. Retinal damage and the neovascularisation process in DR are closely related to the oxidative environment induced by hyperglycaemia. Experimental *in vitro* and *in vivo* studies have provided evidence for the efficacy of polyphenols as potential nontoxic agents to prevent the pathology from advancing. The capability to protect retinas and to avoid the evolution of this disease includes actions mainly against oxidative stress, inflammation, and VEGF production. Given their low bioavailability and concentrations in foods, designing local delivery systems would be a good option to make their use as an effective therapy possible.
